# Combined Impact of a Faster Self-Reported Eating Rate and Higher Dietary Energy Intake Rate on Energy Intake and Adiposity

**DOI:** 10.3390/nu12113264

**Published:** 2020-10-25

**Authors:** Pey Sze Teo, Rob M. van Dam, Ciarán G. Forde

**Affiliations:** 1 Clinical Nutrition Research Centre (CNRC), Singapore Institute of Food and Biotechnology Innovation (SIFBI), Agency for Science, Technology and Research (A*STAR), Singapore 117599, Singapore; Teo_Pey_Sze@sifbi.a-star.edu.sg; 2 Saw Swee Hock School of Public Health, National University of Singapore, Singapore 117549, Singapore; rob.van.dam@nus.edu.sg; 3 Department of Physiology, Yong Loo Lin School of Medicine, National University of Singapore, Singapore 117593, Singapore

**Keywords:** eating rate, energy intake rate, dietary energy intake, adiposity, multi-ethnic cohort

## Abstract

Eating more quickly and consuming foods with a higher energy-intake-rate (EIR: kcal/min) is associated with greater energy intake and adiposity. However, it remains unclear whether individuals who eat more quickly are more likely to consume foods with higher EIR. We investigated the overlap between self-reported eating rate (SRER) and the consumption of higher EIR foods, and their combined impact on daily energy intake and adiposity in a population-based Asian cohort (*n* = 7011; 21–75y). Food consumption was assessed using a validated Food Frequency Questionnaire. Moderated regression with simple slope analysis was conducted to evaluate whether SRER modified the association between dietary EIR and total dietary energy intakes. Faster eaters consumed a significantly higher proportion of energy from higher EIR foods among overweight individuals, but not among normal-weight individuals. Associations between dietary EIR and total energy intake were stronger among medium (β = 15.04, 95%CI: 13.00–17.08) and fast (β = 15.69, 95%CI: 12.61–18.78) eaters, compared with slower eaters (β = 9.89, 95%CI: 5.11–14.67; *p*-interaction = 0.032). Higher dietary EIR also tended to be more strongly associated with BMI in fast eaters (β = 0.025, 95%CI: 0.011–0.038) than in slow eaters (β = 0.017, 95%CI: −0.007–0.040). These findings suggest that the combination of eating more quickly and selecting a greater proportion of energy from higher EIR foods (i.e., softly textured, energy dense), promoted higher dietary energy intakes and adiposity.

## 1. Introduction

Eating at a faster rate is associated with increased acute energy intake [1], and epidemiological studies have consistently shown associations between faster eating, higher body mass and adiposity, and poorer cardio-metabolic health [2]. We recently reported that faster self-reported eating rates (SRER) were associated with a significantly higher energy intake, body weight, body mass index (BMI) and adiposity in a population-based cohort [3]. Differences in eating rates are believed to have a heritable component [4], and the emergence of a faster, ‘obesogenic’ eating style [5] has been shown to be associated with higher fat-free mass, basal metabolic rate and energy requirements [6], and with more rapid weight gain, stronger food approach appetitive traits [7,8] and poorer inhibitory control [9] among children.

Although heritable, an individual’s eating rate is also largely influenced by the oral processing and texture properties of the different foods they choose to consume in their everyday diet [10,11,12]. For example, liquids can be consumed much faster (i.e., with rates of up to 600 mL/min), compared to solid and semi-solid foods. These eating rate differences between foods have been shown to be similar across different food environments [10,11,13,14,15].

A food’s energy intake rate (EIR: kcal/min) is a product of its energy density (kcal/g) and eating rate (g/min), and provides an index of the typical rate at which calories are ingested across different foods [16]. Foods with high EIRs tend to be softly textured, easy to consume quickly, and higher in energy density, enabling a faster energy intake rate (kcal/min). Consuming foods that have higher EIRs (higher kcal/min) is associated with increased energy intake [16], and sustained consumption of higher EIR foods is associated with increased weight gain and adiposity [17]. Cumulatively, the prevalence and frequency of the consumption of higher EIR foods in a diet reflects the energy density and eating rate properties of the foods which an individual habitually chooses to consume, and the availability of fast, easy-to-consume calories in their food environment [15]. The increased consumption of higher EIR foods and beverages within a diet has been linked with higher energy intakes and adiposity in the Singaporean diet [18].

Evidence to date indicates that eating more quickly and choosing to consume foods with a higher EIR are both associated with increased energy intakes and adiposity. It remains unclear the extent to which these behaviors overlap, and what their joint impact is on dietary energy intake and adiposity. The current study investigated the overlap between higher SRER and the consumption of high EIR foods, and their combined impact on daily energy intakes, and measures of adiposity.

## 2. Materials and Methods

### 2.1. Study Population

Cross-sectional data are from the follow-up Singapore Multi-Ethnic Cohort Phase-2 study (MEC2). MEC2 is a population-based cohort study in Singaporean citizens and permanent residents aged 21 to 75 years, comprising of the three major ethnic groups in Singapore: Chinese, Indian and Malay. Detailed information on MEC2 and its follow-up is published elsewhere [19], and can be found at http://blog.nus.edu.sg/sphs/the-first-sphs-follow-up/. Data were included for individuals who participated in both interview and health screening sessions (*n* = 7314), and excluded were those with missing data (*n* = 86), invalid energy intakes (i.e., extreme energy intakes of ≤500 kcal/day or ≥6000 kcal/day) (*n* = 139), and those suffering from major chronic diseases (*n* = 78), (i.e., cancer, heart attack or stroke). A total of 7011 participants were included in the final analysis. Written informed consent was obtained from all participants and the study protocol was approved by the Institutional Review Board of the National University of Singapore (NUS-IRB B-16-125).

### 2.2. Assessment of Self-Reported Eating Rate (SRER), Diet and Body Composition

Data on sociodemographic characteristics, medical history, and dietary and other lifestyle factors were collected through face-to-face interviews by trained staff. Physical activity was assessed using the locally validated SP2 Physical Activity Questionnaire (SP2PAQ) [20], which assesses activity in the leisure, occupational and transport domains. We calculated the total physical activity expressed in metabolic equivalent task units (METs), based on the Ainsworth compendium.

Self-reported eating rate (SRER) was recorded by each individual based on a previously published approach [3,21], and previous research has shown that SRER estimates do reflect experimentally observed eating rates at a group level [22,23]. Each participant self-assessed their eating rate in response to the question: “How fast is your rate of eating?”, where responses were selected from the categories “very slow”, “relatively slow”, “medium”, “relatively fast”, and “very fast”.

Dietary intake data were assessed using a validated semi-quantitative food frequency questionnaire (FFQ) [24] consisting of 163 food items, with additional sub-questions on food sub-types (e.g., types of noodles/rice used), associated ingredients (e.g., added oil and sugar) and cooking methods (i.e., curries with or without coconut, stir-fried, deep-fried, stewed, roasted, and boiled). Participant intake frequencies were recalled over the past year and a trained interviewer provided visual aids to help quantify the standard portion sizes. The relative consumption frequencies of food subtypes and cooking methods in the FFQ were converted to absolute frequencies (servings per day) of the whole dish as consumed. For instance, ‘Fresh chicken’, and ‘In curry with coconut’ were re-coded as a dish: ‘Chicken; curry with coconut’. The reported beverages with extra added sugars were also re-coded as new food items in combination with sugar. This resulted in an extended list of 269 commonly consumed foods from the FFQ.

The eating rate (g/min) of these foods was collated from previously published studies from Singapore, the United Kingdom, Switzerland and the Netherlands [16]. For items not previously measured, their eating rates were matched and imputed from tested foods with similar food forms and/or cooking methods. Eating rates were combined with the energy density of each food item to derive a food’s energy intake rate (EIR: kcal/min), and a summary of the food list with their EIR values is described elsewhere [18].

Details on assessment procedures for body composition have been reported in our previous paper [3]. In brief, body weight, height, and waist-circumference (WC) were assessed according to WHO standards and were taken to the nearest 0.1 kg, 0.1 m, or 0.1 cm, respectively, by trained personnel. Body mass index (BMI) was calculated by dividing weight (kg) by height squared (m^2^). Asian cut-offs for the BMI classification of overweight (≥23 kgm^−2^) were used to identify individuals at moderate risk of obesity-related diseases. Similarly, the WC cut-offs for Asians, i.e., >80 cm for women and >90 cm for men, were used for abdominal obesity.

### 2.3. Statistical Analysis

Descriptive statistics were reported as mean ± SD, unless otherwise indicated. Based on the distribution of the self-reported eating rate (SRER) data, we condensed the two lowest (“very slow” and “relatively slow”) and the two highest (“relatively fast” and “very fast”) SRER categories into two groups “slow” and “fast”, respectively. Dietary EIR for each participant (i.e., the mean EIR across all foods consumed by each individual) was calculated. The calculation was carried out in 3 steps: (1) we first multiplied the EIR of a food consumed (kcal/min) with the amount of the food consumed (g), (2) then totaled the EIR–gram of all foods consumed (g*kcal/min), and (3) divided the sum of EIR–gram of all foods consumed (g*kcal/min) by the total amount of foods consumed (g). The dietary EIR per individual (kcal/min) was then split into dietary EIR tertiles, to divide the population into those with a low, medium, or high EIR of their daily food consumption. Analysis of covariance (ANOVA) was used to determine the differences of all continuous variables across the SRER groups in each of the tertiles of dietary EIR, and Chi-square tests were used to evaluate the differences of the categorical variables across the SRER groups in each of the tertiles of dietary EIR. Moderated regression with simple slope analysis was used to evaluate whether SRER modified the association between (i) dietary EIR and total energy intakes; (ii) dietary EIR and BMI. Interaction between dietary EIR (kcal/min) and SRER (medium/high vs. low) was also tested by including multiplicative interaction terms in the multivariable model.

In addition to the dietary EIR for each individual, we created quartiles for the EIR across all 269 food items. Differences between the foods’ EIR range in the first two quartiles (Q1–low and Q2–medium EIR foods) were small, (i.e., <15 kcal/min), so the first two quartiles were combined into one category, resulting in three categories of EIR foods (i.e., low-to-medium, high and very high EIR foods). Multivariable ANOVA models were used to examine differences in the relative consumption of higher and lower EIR foods across their SRER groups, after adjusting for age (years), sex, ethnicity, education level, physical activity (MET-min/week), smoking and alcohol consumption. This analysis was repeated after stratification by the overweight status of the participants. All statistical analyses were performed using the IBM SPSS for Windows version 26.0 (IBM, Armonk, NY, USA) and *p*-values < 0.05 were considered to be statically significant.

## 3. Results

Table 1 summarizes participant characteristics (N = 7011) for socio-demographic, dietary, lifestyle and body composition profiles according to self-reported eating rate (SRER) and tertiles of dietary energy intake rate (EIR). The largest ethnic group was Chinese (71.1%), followed by Indians (14.7%), Malays (9.0%), and others (5.2%), broadly in line with the population ethnicity distribution for Singapore. The mean age of the participants was 49.8 ± 13.0 years, and 44.8% were male. Most participants were overweight (62.1%) based on Asian BMI cut-offs (≥23 kgm^−2^), with an average BMI of 24.9 ± 4.6 kgm^−2^ (Table 1).

Younger age was associated with both a higher dietary EIR and higher SRER. Higher dietary EIR was associated with higher dietary energy intakes. In addition, dietary energy intakes increased significantly from the slower to faster eaters within each group of dietary EIR (i.e., low, medium and high) (Table 1). Both higher SRER and higher dietary EIR were associated with significantly higher body weight, BMI, and waist-circumference. The combined impact of a higher SRER and higher dietary EIR on greater body mass and waist circumference remained significant (all, *p* < 0.001), after adjusting for socio-demographic and lifestyle factors (data not shown).

**Figure 1:**
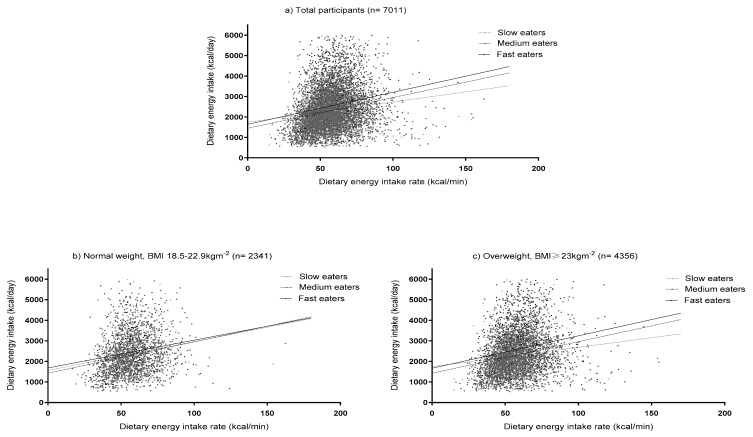
Simple slope analysis representing the modifying effects of self-reported eating rates (SRER) on the association between dietary energy intake rate (EIR) and daily dietary energy intake, among (**a**) total population, and individuals who were (**b**) normal-weight and (**c**) overweight. The three SRER groups represent the rates of eating, i.e., slow, medium and fast.

We examined the association between dietary EIR and total dietary energy intake according to SRER (Figure 1a). The association between dietary EIR and energy intake was stronger among those with medium (β = 15.04, *p* < 0.001, 95%CI: 13.00–17.08) and fast (β = 15.69, *p* < 0.001, 95%CI: 12.61–18.78) eating rates when compared with slow eaters (β = 9.89, *p* < 0.001, 95%CI: 5.11–14.67) (Figure 1a). There were significant interactions between SRER groups and dietary EIR, such that the impact of higher dietary EIR on increased energy intakes was modified by eating at a faster rate; with modifying effects similar for medium and fast eaters (*p*-interaction = 0.032) (Figure 1a). These interactions of SRER groups and dietary EIR remained marginally significant after adjusting for potential confounders (*p*-interaction = 0.068).

In addition, higher dietary EIR tended to be more strongly associated with BMI in fast eaters (β = 0.025, *p* < 0.001, 95%CI: 0.011–0.038) than in medium (β = 0.019, *p* < 0.001, 95%CI: 0.009–0.029) and slow eaters (β = 0.017, *p* = 0.167, 95%CI: −0.007–0.040). After multivariable adjustment, the significant association between dietary EIR and BMI was only observed in fast eaters (β = 0.022, *p* = 0.001, 95%CI 0.009–0.035).

We also evaluated the interaction between dietary EIR and SRER in relation to dietary energy intake after stratification for overweight status (Figure 1b,c). In overweight participants, higher dietary EIR was more strongly associated with energy intake in medium eaters (β = 15.34, *p* < 0.001, 95%CI: 12.73–17.96) and fast eaters (β = 15.77, *p* < 0.001, 95%CI: 12.06–19.49) when compared with slow eaters (β = 9.36, *p* = 0.006, 95%CI: 2.71–16.02) (Figure 1c). This interaction remained significant after multivariable adjustment. No significant interaction was observed between SRER and EIR in relation to energy intake among individuals in the normal-weight range (Figure 1b).

Table 2 shows multivariable-adjusted models for the relative contribution (%) of different EIR foods (i.e., low-to-medium, high and very high) across the SRER groups among those categorized as being overweight, as assessed by both BMI (overweight) and WC (abdominal-overweight). Among overweight individuals (BMI ≥ 23 kgm^−2^), faster eaters consumed a significantly larger percentage of energy from high EIR foods (*p* = 0.003), and a smaller relative intake of low-to-medium EIR foods (*p* = 0.002) when compared with slower eaters. This association was similar in participants who were classed in the abdominal-overweight category. By contrast, no significant associations were observed between eating rate and the contribution of higher EIR foods among non-overweight individuals.

## 4. Discussion

In this population-based cohort study, we observed a synergistic interaction between faster self-reported eating rate (SRER) and the consumption of foods that have a higher energy intake rate (EIR: kcal/min) in relation to higher total dietary energy intakes. This interaction was observed among individuals in the overweight category, but not among those in the normal-weight category. Both higher SRER and higher dietary EIR were associated with a significantly higher body weight, BMI, and waist-circumference, even after multivariable adjustment. Individuals with higher dietary EIR consumed significantly more energy within and across each SRER group, from slow eaters to fast eaters. Overweight individuals who were fast eaters also consumed a significantly greater proportion of their daily energy intake from higher EIR foods, with a lower relative intake of low-to-medium EIR foods. Together, these findings highlight that both higher rates of consumption and consistently selecting foods that had higher EIRs (i.e., softly textured and higher in energy-density) were associated with the highest dietary energy intakes and body mass index. The combination of eating more quickly and selecting higher-EIR foods may be particularly detrimental for excess energy intake.

Eating rate varies between individuals, but is a consistent predictor of the energy intake of individuals when consuming the same foods [25]. Eating at a faster or slower rate may reflect the different individual’s drive to eat, but the current results suggest it may also be associated with selecting foods that can be consumed quickly and that require less extensive mastication. Previous research has highlighted the diversity of energy intake rates in the food environment [15], where non-nutritive properties of a food, such as texture and structure, can impact both the rate and extent of habitual calorie intake. When high energy dense foods are consumed quickly, it results in ‘fast calories’ (e.g., higher EIR foods), which can promote higher energy intakes [10,26]. In this regard, the impact of poor dietary patterns can be further exacerbated by maladaptive eating behaviors, such as faster eating, that are promoted by selecting softly textured foods that can be consumed quickly within a meal [27]. These findings emphasize the need to combine interventions that both slow the rate of consumption using a food’s texture properties, alongside reductions in energy density, and acknowledge the joint contribution of both eating behavior and food composition in promoting excessive energy intakes, particularly among individuals who are overweight and obese.

Eating at a faster rate and selecting energy dense foods that can be consumed quickly can promote increased intake independently, and act in synergy when in combination. This creates opportunities to design interventions that combine changes in dietary choices and eating behaviors to reduce the risk of excessive energy consumption. Previous intervention trials have shown clinically significant reductions in both energy intake and body weight through the use of programs focused on re-training an individual’s eating rate [28,29,30]. There is widespread agreement that the availability of low-cost, energy dense foods that are consumed in large portions can promote passive energy overconsumption [31]. Globally, there are many initiatives that mandate category-wide reductions and upper limits in energy density [32], which have been shown to effectively reduced both acute [26,33,34] and longer-term energy intakes [35]. Opportunities exist to further reduce the risk of excess energy consumption by using a food’s sensory and texture cues to manipulate eating rates [36], and accumulating evidence has shown that enhancing food texture can be used to slow eating rates and reduce energy intake by 10–15% without compromising sensory appeal or post-meal satisfaction [37,38]. Understanding how a food’s sensory properties can be used to encourage slower eating rates and reduce energy density (i.e., lower EIR), affords new opportunities to utilize ‘sensory’ reformulation to reduce both the eating rate and the availability of energy dense foods that are known to promote passive overconsumption.

Mounting evidence suggests that oral processing is associated with postprandial plasma glucose, insulin secretion, and satiety response [39,40,41], which is largely moderated by the food texture and its oro-exposure time. It may be of interest to investigate whether the combined impacts of eating quickly and having a high rate of calorie intake (i.e., high dietary EIR) can be linked to metabolic responses in future studies. In addition, it would be of interest to examine behavioral determinants of differences in eating rates and EIR food preferences among the ethnic groups.

A strength of the current study is in the large multi-ethnic sample population, and the detailed information on potential confounders, including socio-demographic factors and lifestyle practices. However, it is important to acknowledge several methodological limitations. The energy intake and physical activity data were derived from a self-reported FFQ and PAQ, respectively, and may be subject to misreporting or measurement errors. Residual confounding from un-measured or incompletely measured covariates may also have affected the results. Furthermore, the cross-sectional study design limits the causal interference on the combined role of SRER and dietary EIR on changes in energy intake and body composition.

## 5. Conclusions

Our findings suggest that both faster SRER and consuming foods that have a higher EIR may promote higher dietary energy intakes, and are associated with a higher BMI. These findings highlight an opportunity to combine reformulation with behavioral and sensorial approaches to reduce the risk of excess energy consumption. Future longitudinal and experimental studies should consider the effect of reduced eating rate and lower EIR consumption patterns to reformulate both the food environment and the associated eating behaviors.

## Figures and Tables

**Table 1:** Characteristics of participants according to self-reported eating rate (SRER) across the tertiles of dietary energy intake rate (EIR) in the Singapore Multi-Ethnic Cohort 2 (*n* = 7011) ^1^.

	Low Dietary EIR (N = 2337) (43.2 ± 6.0 kcal/min)				Medium Dietary EIR (N = 2337) (56.3 ± 3.2 kcal/min)				High Dietary EIR (N = 2337) (73.1 ± 11.2 kcal/min)			
	Slow Eaters (*n* = 252)	Medium Eaters (*n* = 1430)	Fast Eaters (*n* = 655)	*p*-Trend	Slow Eaters (*n* = 233)	Medium Eaters (*n* = 1354)	Fast Eaters (*n* = 750)	*p*-Trend	Slow Eaters (*n* = 226)	Medium Eaters (*n* = 1363)	Fast Eaters (*n* = 748)	*p*-Trend
Age, years	56.9 ± 12.5 ^ab^ ***	54.0 ± 11.9 ^ab^ ***	51.2 ± 12.5 ^ab^ ***	<0.001	49.5 ± 14.2	50.2 ± 12.6 ^a^ ***	47.0 ± 12.2 ^a^ ***	<0.001	47.9 ± 14.9	47.8 ± 12.9	44.6 ± 12.4	<0.001
Sex, %				<0.001				<0.001				<0.001
Men	52.4	42.8	56.8		38.6	37.9	56.3		33.6	40.1	50.4	
Women	47.6	57.2	43.2		61.4	62.1	43.7		66.4	59.9	49.6	
Ethnic group, %				<0.001				0.002				<0.001
Chinese	71.4	74.9	77.7		71.7	70.4	77.6		62.4	61.3	72.9	
Malay	9.1	9.7	3.5		8.6	8.6	4.0		13.7	14.2	7.5	
Indian	16.3	12.1	14.8		14.2	16.0	14.4		14.6	16.4	14.4	
Others	3.3	3.4	4.0		5.6	5.0	4.0		9.3	8.1	5.2	
Dietary energy intake, kcal/day	2012.8 ± 829.8 ^ab^ ***	1990.5 ± 849.1 ^ab^ ***	2060.9 ± 963.1 ^ab^ ***	<0.001	2468.8 ± 1068.4	2396.7 ± 952.3 ^a^ ***	2669.6 ± 1016.1	<0.001	2470.9 ± 1016.1	2554.2 ± 1062.3	2773.7 ± 1077.5	<0.001
Body weight, kg	62.2 ± 12.5	63.9 ± 12.7 ^a^ ***	67.6 ± 13.7 ^a^ *	<0.001	62.6 ± 15.1	65.0 ± 14.3	69.0 ± 14.4	<0.001	64.2 ± 14.6	65.8 ± 13.9	69.8 ± 15.5	<0.001
BMI, kgm^−2^	23.9 ± 4.4	24.5 ± 4.3 ^a^ **	25.1 ± 4.3	<0.001	23.8 ± 5.0	24.8 ± 4.9	25.2 ± 4.4	0.001	24.8 ± 5.3	25.1 ± 4.8	25.6 ± 4.9	0.021
WC, cm	82.7 ± 11.6	83.3 ± 11.1	85.4 ± 11.3	<0.001	81.2 ± 13.1	83.5 ± 11.9	85.4 ± 11.6	<0.001	83.0 ± 13.2	83.6 ± 11.7	85.5 ± 12.1	0.001

^1^ Unadjusted data. BMI, body mass index; WC, waist circumference. Values are presented as mean ± SD unless otherwise noted. Significant difference from ^a^ high; ^b^ medium dietary EIR of a similar eating rate group with the Bonferroni’s correction at * *p* < 0.05, ** *p* < 0.01, *** *p* < 0.001.

**Table 2:** Multivariable models of differences in relative consumption of higher and lower energy intake rate (EIR) foods among individuals being overweight or abdominally overweight across their self-reported eating rate (SRER) groups in the Singapore Multi-Ethnic Cohort 2 ^1^.

	General Adiposity (N = 7011)								Abdominal Adiposity (N = 7011)							
	Non-Overweight (N = 2655) BMI < 23 kgm^−2^				Overweight (N = 4356) BMI ≥ 23 kgm^−2^				Non-Overweight (N = 4027) WC ≤ 90 cm (Men); ≤80 cm (Women)				Overweight (N = 2984) WC > 90 cm (Men); >80 cm (Women)			
	Slow Eaters (*n* = 343)	Medium Eaters (*n* = 1606)	Fast Eaters (*n* = 706)	*p*-Trend	Slow Eaters (*n* = 368)	Medium Eaters (*n* = 2541)	Fast Eaters (*n* = 1447)	*p*-Trend	Slow Eaters (*n* = 456)	Medium Eaters (*n* = 2366)	Fast Eaters (*n* = 1205)	*p*-Trend	Slow Eaters (*n* = 255)	Medium Eaters (*n* = 1781)	Fast Eaters (*n* = 948)	*p*-Trend
	Mean ± SE				Mean ± SE				Mean ± SE				Mean ± SE			
Percentage of energy intake (%)																
Low to Medium EIR foods (0–40 kcal/min)	48.80 ± 0.72	49.52 ± 0.33	48.89 ± 0.50	0.462	50.14 ± 0.69 ^a^ **	50.30 ± 0.26 ^a^ **	48.80 ± 0.35	0.002	49.04 ± 0.62	49.46 ± 0.27	48.75 ± 0.39	0.313	50.26 ± 0.83 ^a^ **	50.75 ± 0.32 ^a^ **	48.88 ± 0.44	0.003
High EIR foods (40–71 kcal/min)	29.98 ± 0.51	29.59 ± 0.24	29.50 ± 0.36	0.738	28.61 ± 0.48	28.14 ± 0.19 ^a^ **	29.21 ± 0.25	0.003	29.71 ± 0.44	29.23 ± 0.19	29.55 ± 0.27	0.474	28.40 ± 0.58	28.00 ± 0.22 ^a^ *	29.06 ± 0.31	0.018
Very high EIR foods (72–300 kcal/min)	21.23 ± 0.55	20.89 ± 0.25	21.61 ± 0.38	0.298	21.25 ± 0.53	21.53 ± 0.20	22.03 ± 0.27	0.246	21.25 ± 0.47	21.31 ± 0.21	21.71 ± 0.29	0.504	21.34 ± 0.64	21.27 ± 0.24	22.06 ± 0.33	0.160

^1^ Adjusting for age (years), sex, ethnicity, highest education level, smoking status and alcohol drinking status, and total PA (METmin/week). Significant difference from ^a^ fast eaters with the Bonferroni’s correction at * *p* < 0.05, ** *p* < 0.01.
